# *KRAS* mutation in secondary malignant histiocytosis arising from low grade follicular lymphoma

**DOI:** 10.1186/s13000-018-0758-0

**Published:** 2018-10-15

**Authors:** Sarah M. Choi, Aleodor A. Andea, Min Wang, Amir Behdad, Lina Shao, Yanming Zhang, Xinyan Lu, David Dittmann, Juan Castro, Yi-Hua Chen, Juehua Gao

**Affiliations:** 1 Department of Pathology, Northwestern University Feinberg School of Medicine, 251 E Huron Street, Chicago, IL 60611 USA; 2 Current address: Department of Pathology, University of Michigan, 5242 Medical Science Building 1, 1301 Catherine Street, Ann Arbor, MI 48109 USA; 3 Current address: Department of Pathology, Memorial Sloan Kettering Cancer Center, 1275 York Ave, New York, NY 10065 USA; 4 Diagnostic Molecular Biology Laboratory, Northwestern Memorial Hospital, 251 E Huron Street, Chicago, IL 60611 USA

**Keywords:** KRAS, Transdifferentiation, Langerhans cell sarcoma, Follicular lymphoma

## Abstract

**Background:**

Transformation of follicular lymphoma most typically occurs as diffuse large B-cell lymphoma, however other forms of transformation such as classic Hodgkin lymphoma and lymphoblastic transformation can occur. Secondary malignant histiocytosis also represents a rare form of transformation, which is thought to occur due to a process of transdifferentiation whereby the lymphoma cells exhibit lineage plasticity and lose all evidence of B-cell phenotype and instead acquire the phenotype of a histiocytic neoplasm. Little is known about the underlying genetic alterations that occur during this unusual process. Comparative genetic analysis of pre- and post-transformation/transdifferentiation would be one tool by which we could better understand how this phenomenon occurs.

**Case presentation:**

Here we report the clinical, immunophenotypic and genetic features of a rare case of secondary malignant histiocytosis, Langerhans cell-type (Langerhans cell sarcoma) arising from a previous low grade follicular lymphoma. FISH analysis confirmed the presence of *IgH/BCL2* rearrangement in both the low grade follicular lymphoma (FL) and transformed Langerhans cells sarcoma (LCS) samples, demonstrating a clonal relationship. Comparative whole exome sequencing was then performed, which identified a *KRAS* p.G13D mutation in the LCS that was not present in the FL.

**Conclusions:**

This report highlights genetic alterations, in particular an acquired somatic *KRAS* mutation, that may occur during transdifferentiation, with additional significance of *KRAS* mutation as a possible therapeutic target in cases which otherwise would have limited treatment options.

**Electronic supplementary material:**

The online version of this article (10.1186/s13000-018-0758-0) contains supplementary material, which is available to authorized users.

## Background

Follicular lymphoma (FL) is an indolent B-cell lymphoma composed of follicle center B cells [1]. While generally regarded as a manageable disease with a long survival rate, it can also progress to more aggressive forms of lymphoma, such as diffuse large B-cell lymphoma. Another rare and distinct form of disease progression is transdifferentiation from a B cell neoplasm to a neoplasm of another cell lineage [2–9]. In these instances, the neoplastic cells demonstrate lineage plasticity, manifested as an apparent shift in cellular phenotype from one lineage to another while still carrying shared underlying clonal genetic abnormalities. Most reported cases have involved transdifferentiation of chronic lymphocytic leukemia to histiocytic neoplasms [2–4, 6, 8, 9]. Transdifferentiation is an elusive process, and the exact pathogenic mechanisms remain unknown. Comprehensive genetic analysis could provide a useful tool by which to identify genetic alterations that occur during transdifferentiation and may provide clues to the underlying pathogenesis of this process. Furthermore, as this form of transformation heralds a very poor prognosis, identification of targetable genetic mutations could offer therapeutic options in cases where there are few alternatives. Here, we described a rare occurrence of a low grade FL transdifferentiating into a Langerhans cell sarcoma (LCS) with acquisition of *KRAS* mutation. As comparative whole exome sequencing of pre- and post-transdifferentiation lymphoma samples has not yet been reported in the literature, this report will contribute to the presently limited understanding of this rare phenomenon.

## Case presentation

Our patient had a long history of follicular lymphoma which initially presented as a localized neck mass. A biopsy of the mass showed low grade follicular lymphoma. The patient was treated with radiation therapy, but 3 years later, was diagnosed with diffuse large B cell lymphoma involving lung with discordant low grade follicular lymphoma in the marrow. After eight cycles of R-CHOP therapy (rituximab, cyclophosphamide, doxorubicin, vincristine, and prednisone), the patient went into remission. Unfortunately, 6 years later, the patient presented with skin and breast lesions as well as increased adenopathy, and was treated with rituximab, with some improvement of adenopathy. The skin nodules on the left arm were noted enlarging, a subsequent biopsy revealed low-grade follicular lymphoma, which responded to rituximab and bendamustine therapy. A few months later, the patient noted an enlarged inguinal lymph node, a biopsy of which showed Langerhans cell sarcoma. Despite 4 cycles of ICE chemotherapy and 8 weeks of ibrutinib, a PET/CT showed evidence of disease progression with diffuse intensely hypermetabolic soft tissue nodules and lymph nodes. Biopsy of one of the lymph nodes was consistent with diffuse large B cell lymphoma. The patient received palliative radiation therapy and passed away in a hospice facility. The patient’s clinical history and treatment was summarized in Table 1.

**Table 1:** Summary of Clinical, Pathologic and Genetic Characteristics of Tumors

Timeline	Location	Diagnosis	Immunophenotype of Neoplastic Cells	Key FISH/Molecular Characteristics	Therapy
Year 1	Neck mass	FL (Fig. 1)	CD19+, CD10+, CD5-, surface lambda restricted	NA	Radiation
Year 3	Lung and bone marrow	Lung: DLBCL Bone marrow: FL (Fig. 2)	Lung: CD20+, CD10+ Bone marrow: CD19+, CD10+, CD5-, surface lambda restricted	NA	R-CHOP
Year 9	Skin and breast	FL (Fig. 3)	CD20+, CD10+, BCL6+, BCL2+	*IgH/BCL2* fusion *KRAS* wildtype *MYC* extra copies; no *MYC* rearrangement	R-Bendamustine
Year 10	Inguinal lymph node	LCS (Fig. 4)	CD45+, CD43+, CD1a+, S100+, CD20-, PAX5-, CD19-, CD79a-, CD10-, BCL6+ (focal)	*IgH/BCL2* fusion *KRAS* p.G13D *MYC* extra copies; no *MYC* rearrangement	ICE, ibrutinib
Year 10	Ear mass	DLBCL	CD20+, CD10+, CD1a-, S100-	NA	Palliative radiation therapy

*FL* Follicular Lymphoma, *LCS* Langerhans Cell Sarcoma, *DLBCL* Diffuse Large B cell Lymphoma, *NA* Tissue Not Available

### Histology

**Fig. 1:**
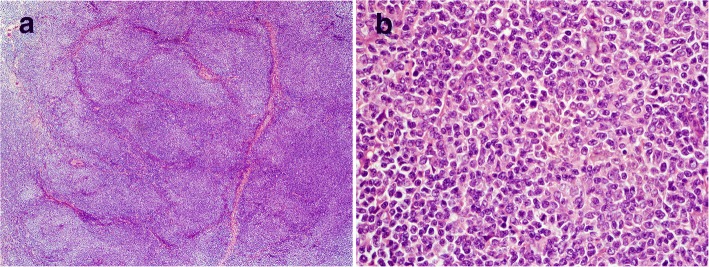
Diagnostic low grade follicular lymphoma. Excisional biopsy reveals a nodular lymphoid proliferation of small lymphocytes with elongated nuclei (**a** H&E, 100X; **b** H&E, 600X). Flow cytometric analysis identifies a surface lambda light chain-restricted B-cell population that is CD10+ and CD5-

The initial biopsy of the mass at presentation showed complete architectural effacement by a nodular lymphoid proliferation of small lymphocytes with elongated and cleaved nuclei (Fig. 1). Flow cytometric analysis identified a surface lambda light chain-restricted B-cell population that was CD10+ and CD5-. Diagnosis of a low grade follicular lymphoma was made.

**Fig. 2:**
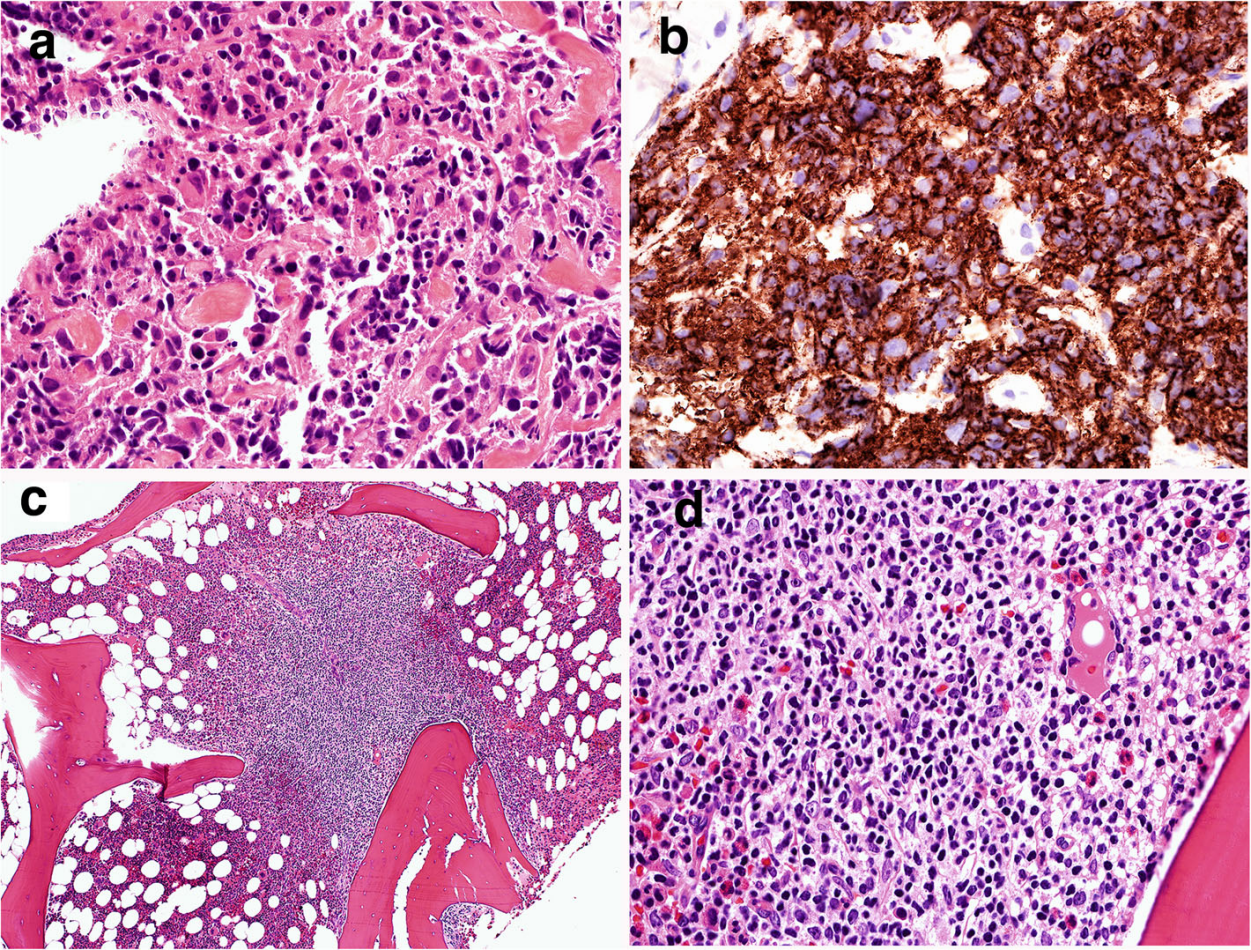
Diffuse large B-cell lymphoma. A transbronchial biopsy of the lung mass shows a diffuse proliferation of CD20+ large cells with large nuclei, open chromatic, occasional nucleoli associated with frequent apoptotic bodies (**a** H&E, 600X; **b** CD20 IHC, 600X). A bone marrow core biopsy shows an abnormal paratrabecular lymphoid infiltrate comprising of small lymphocytes with elongated nuclei (**c** H&E, 200X; **d** H&E, 600X). Flow cytometric analysis of the bone marrow aspirate (not shown) identified a monotypic, surface lambda light chain-restricted B-cell population that was dim CD10+ and CD5-, consistent with bone marrow involvement by follicular lymphoma

Three years later, a transbronchial biopsy showed atypical mononuclear cell infiltrate composed of large cells with large nuclei and prominent nucleoli associated with frequent apoptotic bodies (Fig. 2). Immunohistochemical staining showed the large cells were positive for CD20 and CD10, and exhibited a moderate proliferation rate based on Ki-67 staining (40–50%). These findings were consistent with diffuse large B-cell lymphoma. A concurrent bone marrow biopsy showed a paratrabecular lymphoid infiltrate composed of small centrocytes consistent with involvement by follicular lymphoma (Fig. 2). Flow cytometric analysis identified a monotypic, surface lambda light chain-restricted B-cell population that was dim CD10+ and CD5-, supporting involvement by follicular lymphoma.

**Fig. 3:**
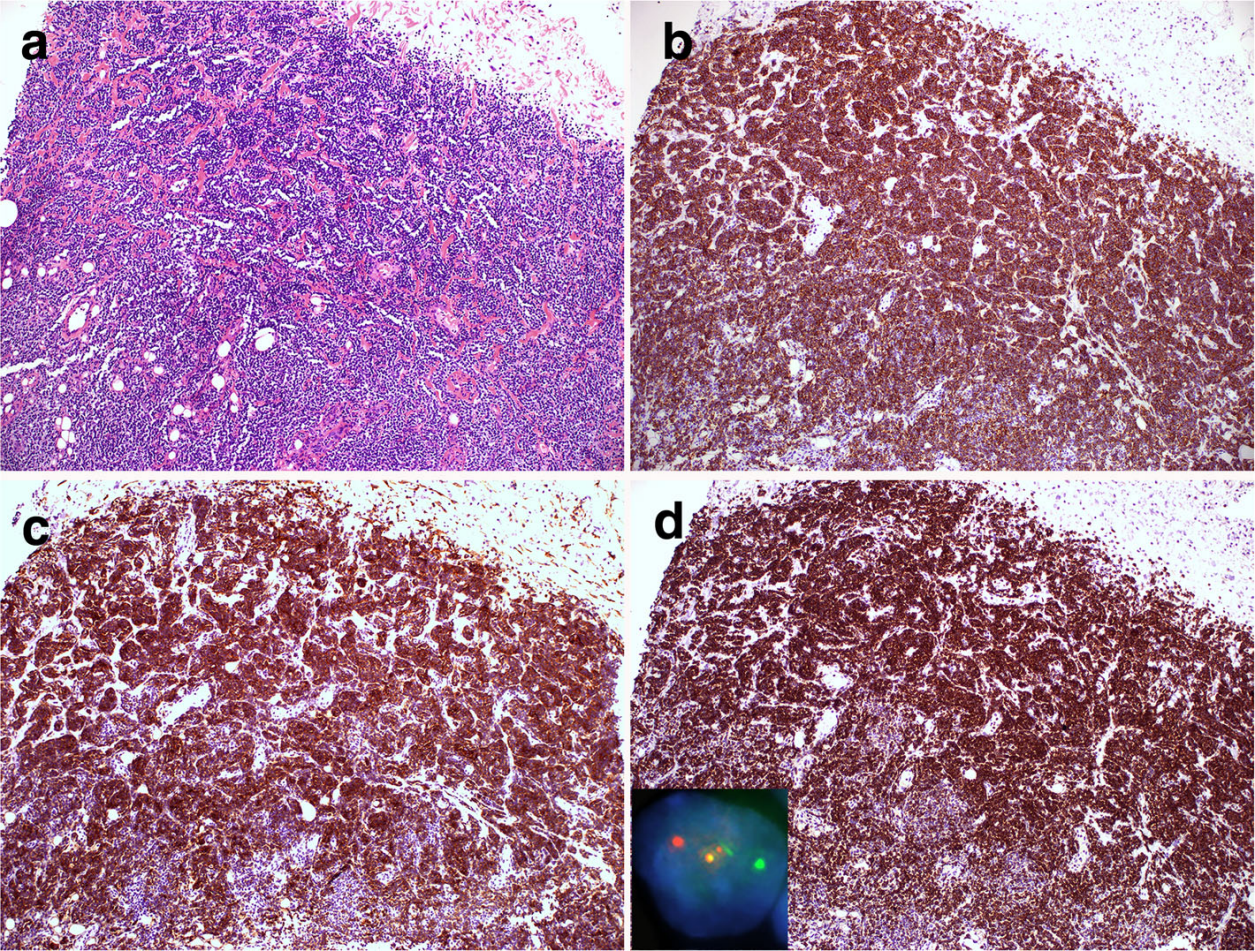
Recurrent low grade follicular lymphoma**.** A needle core biopsy reveals an abnormal infiltrate of small lymphocytes that are predominantly CD20+ B cells with coexpression of CD10, BCL6 and BCL2, consistent with low grade follicular lymphoma (**a** H&E, 200X, **b** CD20 IHC, 200X; **c** CD10, 200X, **d** BCL2 IHC, 200X). Dual color dual fusion FISH analysis confirms the presence of *IgH/BCL2* fusion signals resulting from t(14;18) in 62% of cells analyzed (Inset)

**Fig. 4:**
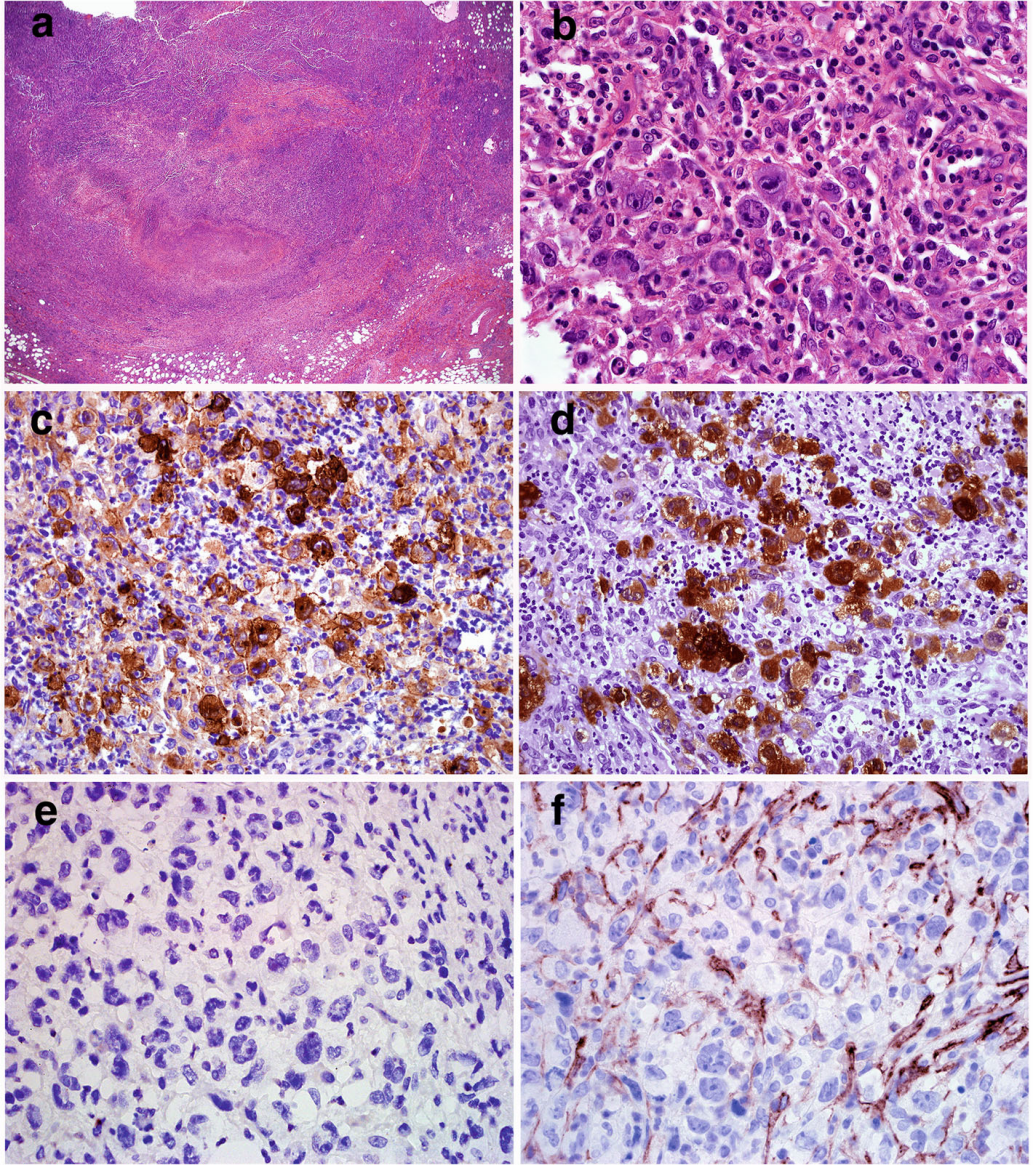
Langerhans cell sarcoma. The inguinal lymph node biopsy shows a complete effacement of the lymph node architecture by an abnormal polymorphous infiltrate of histiocytes, small lymphocytes, neutrophils, eosinophils and rare plasma cells, with scattered and focal sheets of atypical large cells with convoluted nuclei (**a** H&E, 100X, **b** H&E, 600X). The large cells are CD1a + and S100+ (**c** CD1a IHC, 600X; **d** S100 IHC, 600X). The morphology and phenotype is consistent with a diagnosis of Langerhans cell sarcoma. The large cells were CD20-, CD19- (not shown), PAX5- (not shown) and CD10- (**e** CD20 IHC, 600X; **f** CD10 IHC, 600X)

The breast biopsy showed an abnormal lymphoid infiltrate composed predominantly of small lymphocytes with elongated nuclei and condensed chromatin. The abnormal lymphocytes were CD20+, CD10+, BCL-6+ and BCL-2+, consistent with recurrent follicular lymphoma (Fig. 3). A few months later, an enlarged inguinal lymph node showed effacement of architecture by an abnormal polymorphous infiltrate composed of many highly atypical large cells with admixed histiocytes, small lymphocytes, neutrophils, eosinophils and rare plasma cells (Fig. 4). The large pleomorphic cells had deeply convoluted nuclei and abundant cytoplasm. Immunohistochemical stains showed the large cells were positive for CD45, CD43, CD1a, and S-100. CD20 and PAX5 immunostains showed virtually no B cells present in the lesion. Flow cytometric analysis did not identify a distinct CD19+ B-cell population. The findings were consistent with Langerhans cell sarcoma.

### FISH analysis

**Fig. 5:**
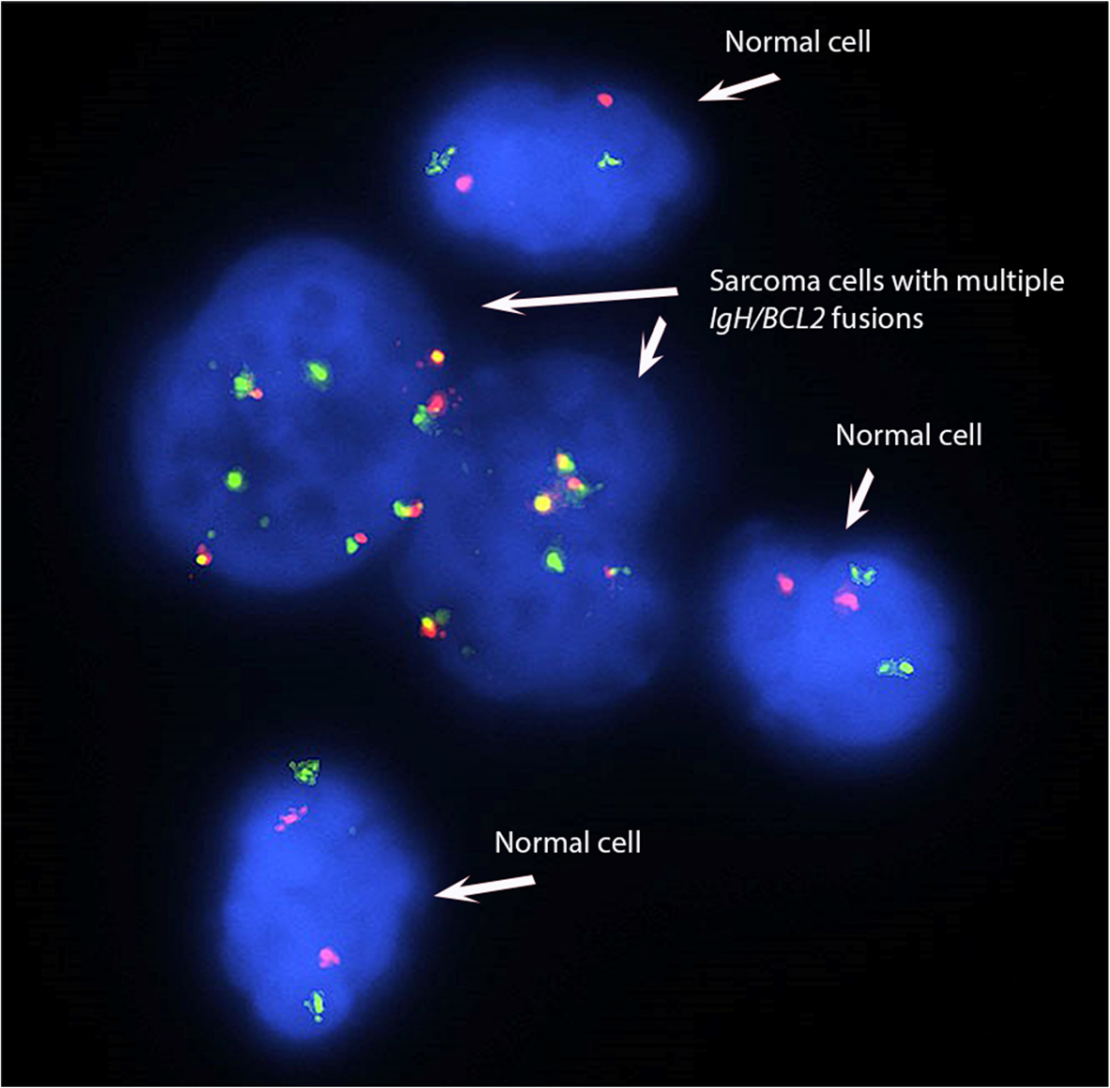
Dual color dual fusion FISH analysis demonstrates multiple *IgH/BCL2* fusion signals resulting from t(14;18) in 21% of cells analyzed. The fusion signals are present only in the large sarcoma cells with but not in any of background inflammatory cells

To establish the genetic relationship between the LCS and the patient’s prior FL, FISH analysis for t(14;18) *BCL2/IgH* was performed on the touch imprints of the recurrent breast follicular lymphoma and LCS. FISH analysis revealed the presence of t(14;18) *BCL2*/*IgH* fusion signals in 62% and 21% of the 200 cells analyzed in follicular lymphoma and LCS respectively (Fig. 3 and Fig. 5). In LCS, the fusion signal is only present within the sarcoma cells identified by their enlarged size and bizarre nuclear morphology, but not in any background inflammatory cells. And almost all the sarcoma cells harbor this translocation. Interestingly, the sarcoma cells displayed 4–5 copies of *IgH*/*BCL2*, indicating amplification of fusion signals from t(14:18) (Fig. 5). Using *MYC* break apart probes, both FL and Langerhans cell sarcoma sample were negative for *MYC* rearrangement; however, extra *MYC* signals were seen in 21% and 22% of the 200 cells analyzed in FL and LCS respectively, and in latter only present in the sarcoma cells. Molecular analysis was performed on the bone marrow aspirate with involvement of follicular lymphoma. The result was positive for clonal IGH gene rearrangement with 334 bp, 273 bp and 133 bp clonal bands by FR1, FR2 and FR3 biomed primers. Molecular analysis was also performed on the LCS and was negative for clonal immunoglobulin heavy chain rearrangement.

### Molecular analysis and whole exome sequencing

Additional molecular analysis for *BRAF* V600E mutation, which has been reported in 38–57% of Langerhans cell histiocytosis but not as of yet in any cases of Langerhans cell sarcoma [1, 10, 11], and was negative for this mutation.

Comparative whole exome sequencing was performed on paraffin-embedded tissue sections from the breast low-grade follicular lymphoma sample and Langerhans cell sarcoma sample. The tumor burden was estimated at 90% and 20–30% with median depth of read of 140 and 92 for the follicular lymphoma and Langerhans cell sarcoma samples, respectively. After filtering, there were 12 variants including 10 nonsynonymous SNVs and two inframe mutations shared by both the FL and LCS. The majority of SNVs, which had a variant allele frequency (VAF) close to 0.5 in both samples or had alternative allele frequency above or close to 0.01 in the 1000 g or ExAC database, may represent germline polymorphisms (Table 2, Additional file 1: Table S1). The shared *CREBBP* mutation (NM_001079846:exon29:c.4920_4922del:p.1640_1641del) has a VAF lower than other shared variants (0.31 in FL and 0.23 in LCS), and may represent a shared somatically acquired driver mutation. CREBBP is a histone modifier that is frequently mutated in  FL [12, 13].

**Table 2:** Summary of Variants with Possible or Unknown Significance Present in Follicular Lymphoma or/and Langerhans Cell Sarcoma

Variants Shared in FL and LCS	Variants present only in FL	Variants present only in LCS
10 nonsynonymous SNVs *SPEN* p.K1064I *PDE4DIP* p.R1867C *PDE4DIP* p.E573V *PDE4DIP* p.S438 L *PDE4DIP* p.A127T *ARHGAP26* p.R293W *PMS2* p.I18V *ATM* p.S49C *KMT2D* p.P1131L *SPECC1* p.D688N	4 nonsynonymous SNVs *IL2* p.V89 L *CDKN2A* p.M1T *BCL2* p.S203 N *BCL2* p.A45T *SMARCA4* p.G883D	11 nonsynonymous SNVs *BIN1* p.H283Y *TMEM200A* p.G383R *DNAJB12* p.R13W *KRAS* p.G13D *TRPV42* p.R249H *ADAD2* p.R299Q *KRTAP4–7* p.S113C *EMR2* p.C520F *ARMCX4* p.G1814E *ARMCX4* p.E1816G *ARMCX4* p.G1820E
2 inframe deletions *ZNF384* p.385_385del *CREBBP* p.1640_1641del	1 stopgain *KMT2D* p.R5097X	2 frameshift insertions *CAMTA1* p.N1271 fs *BCR* p.G1049 fs
	3 inframe deletions *MLLT3* *MN1* p.524_524del *MN1* p.304_304del	2 inframe deletions *ARID1A* p.1333_1334del *NOTCH1* p.2411_2411del

Twelve variants were present in the FL but not in the LCS. After filtering, there were 4 remaining nonsynonymous SNVs, 1 stopgain, and 3 inframe deletions (Table 2, Additional file 2: Table S2)*.* Included among these variants were *KMT2D* p.R5097* and *BCL2* p.S203 N, which have been previously identified in FL [14, 15].

Although copy number alterations were not covered in this assay, analysis of allele frequency of commonly occurring SNVs can be a useful indicator of loss of heterozygosity (LOH). There were 14 nonsynonymous single nucleotide variants demonstrating potential LOH (i.e. heterozygous in the FL and homozygous in the LCS). None of the variants were considered deleterious (Table 2, Additional file 3: Table S3).

**Fig. 6:**
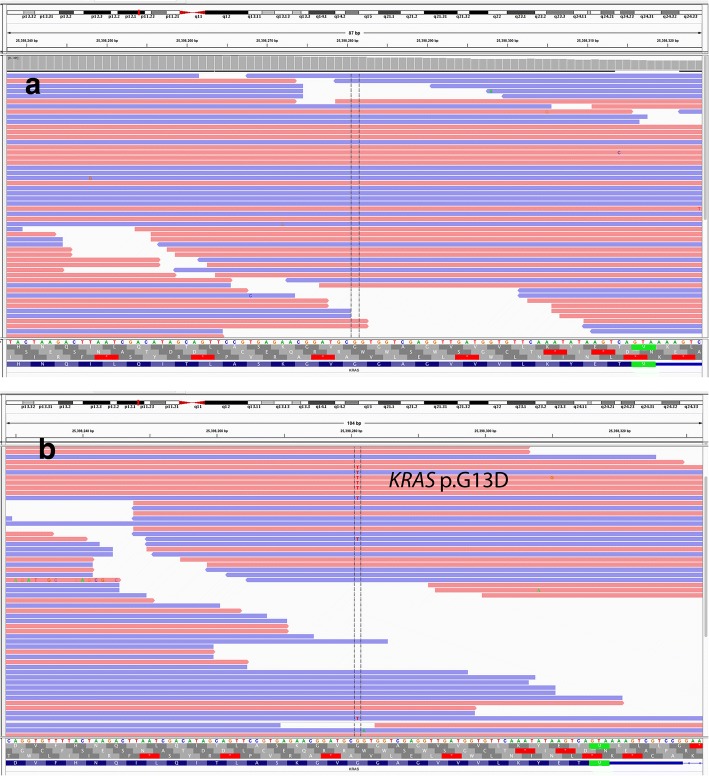
Exome sequencing performed on the Langerhans cell sarcoma sample revealed a G13D mutation results in an amino acid substitution at position 13 in *KRAS*, from a glycine to an aspartic acid (Variant allele frequency: 0.24) (**b**). This *KRAS* p.G13D mutation was not present in the previous follicular lymphoma (**a**)

Thirty variants were identified in the LCS sample that were not present in the FL sample including 26 nonsynonymous SNVs, 2 frameshift insertions and 2 inframe deletions. After filtering out of benign or likely benign variants, the remaining variants included 11 nonsynonymous SNVs, 2 frameshift insertions and 2 inframe deletions (Table 2, Additional file 4: Table S4). Most of the variants were of unknown significance, except *KRAS* p.G13D (Fig. 6), which is a well-known pathogenic variant in a variety of malignancies including carcinoma and hematopoietic neoplasms [16–18]. To confirm the presence of *KRAS* mutation, a real-time PCR analysis was also performed in both the FL and LCS. The results confirmed the presence of *KRAS* p.G13D in the LCS but not in the FL (data not shown).

### SNP microarray analysis

Multiple chromosomal abnormalities were identified in the follicular lymphoma sample which include 6p CN-LOH, 6q deletion, small gain at 7p, 9p21.3 deletion (CDKN2A/2B genes included), small deletion at 10q, small deletion at 12q (distal breakpoint within BCL7A gene), 16p deletion, small 16p gain, 2 CN-LOHs on 20p and some gains involving 20p, a homozygous loss involving chromosome 22q11.22 involving the IGL gene and likely related to gene rearrangements in the tumor, a CNV gain involving chromosome 22q11.23 and gain of entire chromosome X (Table 3). However, there were no significant abnormalities identified in the Langerhans cell sarcoma sample with the exception of *KRAS* p. G13D and a CNV gain on chromosome 22q11.23, which was also observed in the follicular lymphoma sample (Table 3).

**Table 3:** Chromosomal abnormalities in Follicular lymphoma and Langerhans cell sarcoma detected by SNP microarray

Sample	Type	Chromosome	Cytoband	Size (Mb)	Comment
Follicular Lymphoma	Loss	6	q11.1-q27	109.02	
		9	p21	0.74	CDKN2A gene
		10	q24.1	1.43	
		16	p13.3-p13.12	14.25	
	Homozygous loss	22q	q11.22	0.5	IGL gene
	Gain	7	p21.1-p15.3	4.18	
		16	p11.2	1.01	
		20	p12.2-p12.1	4.52	
		20	p11.23-q13.33	44.31	
		X	p22.33-q28	155.27	Whole chromosome gain
	High copy gain	22	q11.23	0.05	CNV
	CN-LOH	6	p25.3-p11.1	58.73	
		20	p13-p12.2	9.45	
		20	p12.1-p11.23	5.06	
Langerhans cell sarcoma	Point mutation	12	p12.1	–	p.G13D c.38G > A
	High copy gain	22	q11.23	0.05	CNV

## Discussion and conclusions

Classification of hematopoietic neoplasms is predicated on the assumption that the phenotype of a neoplastic cell is intrinsically linked to a predefined lineage and that this lineage determines the type and nature of the neoplasm. However, as demonstrated in cases of transdifferentiation, these associations are not hard and fast rules, as cells, both physiologic and neoplastic, can demonstrate lineage plasticity [3–9, 19–21]. Our patient’s pathologic studies include a wide range of morphologic variation in the neoplastic clone, ranging from low grade FL to DLBCL to later on, LCS.

The mechanisms by which transdifferentiation occurs are largely unknown and previously described cases have relied upon molecular clonality or other genetic markers to establish a clonal relationship between what otherwise would appear as two phenotypically distinct neoplasms. Clonal *IgH* rearrangements have been reported in 39% of sporadic cases of histiocytic/dendritic cell sarcomas [22] which may reflect the close relationship and possible common precursor between neoplasms of histiocytic differentiation and lymphoid differentiation [4, 6, 7, 9, 19, 23–25]. In our case, while a clonal *IgH* rearrangement was detected in the FL from the fresh bone marrow sample, PCR analysis failed to identify a clonal *IgH* rearrangement in LCS. The reason for the absence of clonal *IgH* rearrangement in the LCS sample is not entirely clear, but could be due to additional genetic alterations affecting the specific binding of the PCR primers or decreased assay sensitivity in formalin fixed tissue. However, the clonal relationship between FL and LCS was definitively confirmed by FISH analysis, which able to readily identify the t(14;18), *IgH/BCL2* within morphologically-apparent sarcoma cells. The shared t(14;18), *IGH/BCL2* translocation in both the follicular lymphoma and LCS by FISH provided strong evidence of their clonal relationship, as this translocation is considered very specific for follicular lymphoma and has not been reported in any de novo sarcoma.

The shared *CREBBP* mutation (NM_001079846:exon29:c.4920_4922del:p.1640_1641del) is an interesting finding. *CREBBP* mutations, particularly mutations of the acetyltransferase domain, has been implicated as an early driver event in follicular lymphoma [26, 27]. Although the specific *CREBBP* mutation that we have detected in this case has not been previously reported in COSMIC database, it does occur within this functional acetyltransferase domain (p.1342-p.1649) [28, 29]. Thus, it could potentially represent a shared acquired somatic driver mutation and further support a model of direct transdifferentiation of LCS from antecedent follicular lymphoma rather than, for example, evolution from a common precursor neoplastic cell harboring an early translocation t(14;18) event.

The additional SNP microarray results also suggest that the LCS likely originated from an earlier follicular lymphoma with t(14;18). As the LCS does not share the other complex genetic alterations with the exception of the shared CNV gain involving 22q11.23. However, it is worth pointing out that the LCS sample contains relatively low tumor burden (20–30% by visual estimation) with a dense background of inflammatory cells, whereas the follicular lymphoma contains almost 100% tumor cells. Therefore, other more subtle genetic alterations in the sarcoma cells may not be identified by the OncoScan SNP microarray which requires at least 25% tumor content.

The most intriguing finding in this case is the detection of *KRAS* p. G13D, a known pathogenic mutation, detected solely in the LCS sample. *RAS* mutations have been implicated in a number of hematologic malignancies, with *NRAS* mutations being more prevalent than *KRAS* mutations, particularly in myeloid neoplasms [30]. RAS-MAPK alterations have also been frequently described in hairy cell leukemia and multiple myeloma, but are more rarely seen in other lymphoproliferative disorders, such as diffuse large B-cell lymphoma [31, 32]. In studies analyzing mutational profiles of follicular lymphoma, a pathogenic role for *RAS* mutation in the development of follicular lymphoma has not been described [33, 34], although it has been reported as a rare event in cases of histologic transformation [35]. In a study of 55 primary DLBCL samples, *KRAS* p.G13D mutation was identified in 2 cases, thus is it possible that *RAS* mutations could drive the pathogenesis of a relatively rare subset of primary and secondary DLBCL [32]. A separate study described differential gene expression of genes in the MAPK signaling pathway, including *NRAS*, in cases of follicular lymphoma compared with transformed follicular lymphoma, suggesting activation of this pathway could be important in disease transformation [36]. In our case, the *KRAS* p.G13D may have arisen during the transformation process. Further studies to completely delineate clonal derivation were desired but precluded by the absence of material from original FL and subsequent DLBCL for additional testing. Nonetheless, the high prevalence of *KRAS* p.G13D mutation in the sarcoma cells, inferred from a variant allele frequency similar to the sarcoma tumor burden, suggests that it is a predominant mutation within the sarcoma cells.

Our finding raises a couple of important questions. What is the significance of *KRAS* mutation in histiocytic neoplasms? Could this mutation potentially drive transdifferentiation process itself? A recent abstract presentation [37] retrospectively reviewed cases of histiocytic sarcomas and discovered *RAS/MAPK* mutations in 44% (*n* = 8) of cases. *KRAS* p.G13D was present in 3 of the cases. Some of the cases of histiocytic sarcoma also possessed mutations that are common in B-cell lymphoma (such as *MYD88, SOCS1, KMT2D, ARID1A)* and furthermore, a subset of patients with histiocytic sarcoma had concurrent or prior history of B-cell lymphoma, including follicular lymphoma, which raised the possibility of transdifferentiation. This suggests that *RAS/MAPK* mutations are actually quite common in histiocytic neoplasms, even some that may be secondary to B-cell lymphoma, and that given their prevalence, may actively participate in driving this disease process.

In terms of Langerhans cell neoplasms, the *RAS/MAPK* signaling pathway has been strongly implicated as a pathogenic driver. Recent mouse models have demonstrated that expression of *KRAS* p.G12D mutation in lung myeloid cells induce the development of pulmonary Langerhans cell histiocytosis (LCH) [38]. *NRAS* p.Q61K mutations have also been reported at higher frequency in cases of pulmonary LCH [39], but have not thus far been seen in non-pulmonary cases. A study of 61 cases of non-pulmonary LCH reported *BRAF* V600E mutations in half of cases, also detected several other point mutations including *TP53* p.R175H, *KRAS* p.G13D and *MET* p.E168D [10]. BRAF is an essential component of the RAS-RAF-MEK-ERK signaling cascade, which is triggered by binding of extracellular growth factor or cytokines to surface tyrosine kinase receptors, eventually leading to the modulation of downstream gene expression [40]. The activation of this pathway in LCH is further supported by the fact that MEK and ERK phosphorylation could be detected by immunohistochemistry in all cases of LCH, regardless of *BRAF* mutational status [41]. Activating mutations in *BRAF* other than V600E have also been detected in LCH [42–44]. Furthermore, cases without *BRAF* V600E mutation have demonstrated a high prevalence of somatic *MAP2K1* mutation, which are mutually exclusive with *BRAF* mutations [43, 45]. Thus, there is a substantial amount of evidence to suggest that the RAS-RAF-MEK-ERK signaling pathways is important to the evolution of LCH.

LCS is an extremely rare malignancy thought to be derived from Langerhans histiocytes, and because of the scarcity of reported cases, the molecular pathogenesis of this entity is mostly unknown. However, it is not unreasonable to infer that some of the same pathways that drive LCH could also drive LCS. Interestingly, a recent report of histiocytic sarcoma transdifferentiation from B-lymphoblastic leukemia demonstrated *NRAS* p.G12D mutation, further suggesting RAS pathway alterations may be important in the transdifferentiation process [46]. It is also possible that other genetic or epigenetic changes which are as yet uncharacterized may contribute to lineage conversion and phenotypic switch seen during transdifferentiation.

Because of its prevalent role in neoplasms of multiple different organ systems, the RAS-RAF-MEK-ERK signaling pathway is also a target for novel therapeutics, including monoclonal antibodies and small molecule inhibitors, for example BRAF and MEK inhibitors in melanoma [47–49]. KRAS is one integral component of the RAS-RAF-MEK-ERK signaling pathway that acts upstream of BRAF [50]. Detection of *KRAS* mutation is now recommended in colorectal carcinoma, since its presence indicates resistance to targeted therapy against upstream signaling proteins (i.e. EGFR) [51]. Recent studies have shown specific efficacy of cetuximab in colorectal cancer patients with *KRAS* p.G13D mutations (the same mutation detected in our patient) and not other *KRAS* mutations [52, 53]. Ongoing studies in a variety of neoplasms examining RAS proteins and other downstream mediators, such as BRAF and MEK, as druggable targets offer additional therapeutic possibilities in patients with applicable genetic alterations, such as the current case [54–56].

It is important to recognize that while the results of whole exome sequencing in this case suggest complex genetic reprogramming may occur during the transdifferentiation process, this method may fail to identify all possible genetic abnormalities, including splice variants or chromosomal gains/losses. As such, additional genetic events may play a cooperative role in developing this aggressive disease. However, the detection of an activating *KRAS* mutation, which is not typically associated with B-cell lymphoma, combined with the growing body of evidence that this signaling pathway is essential to the development of Langerhans cell neoplasms, suggests that acquisition of this mutation could potentially drive or influence transdifferentiation.

In summary, this case describes transformation/transdifferentiation of a low grade FL into a LCS with detection of *KRAS* p.G13D mutation in the transformed sample. Our findings demonstrate characterize genetic events that may occur during transdifferentiation and further emphasizes the role of alterations of RAS-RAF-MEK-ERK signaling in the pathogenesis of Langerhans cell neoplasms. Importantly, the discovery of acquired *KRAS* mutation raises the possibility of targeted therapies (e.g. small molecule inhibitors) [50, 57] in the treatment of patients with these neoplasms who otherwise have limited therapeutic options.

## Materials and methods

### Pathology review

H&E sections from the patient’s previous biopsies were retrieved from the Department of Pathology at Northwestern Memorial Hospital. The available archival materials were reviewed, along with associated ancillary studies, including immunohistochemical stains performed at the time of diagnosis, flow cytometric analysis and molecular analysis. Additional immunohistochemical stains, molecular and fluorescence in situ hybridization (FISH) analysis were performed in this study and are further described.

### Immunohistochemistry

Immunohistochemical staining was performed on formalin-fixed, paraffin-embedded tissue. Antigen retrieval and immunohistochemical stain were performed on an automated immunostainer following the manufacturer’s protocol (Ventana Medical Systems, Tuczon, AZ). The following predilute antibodies were used: CD3 (Ventana), BCL-2 (Cell Marque, Rocklin, CA), BCL-6 (Cell Marque), Ki-67 (Ventana), CD43 (Ventana), CD1a (Beckman Coulter, Miami, FL), S-100 (Ventana). The following antibodies were also used: CD20 (Dako, Carpinteria, CA), Pax-5 (1:10, Cell Marque), CD10 (1:30, Leica, Buffalo Grove, IL), CD45 (1:300, Dako). Positive and negative controls were performed with all cases and showed appropriate staining.

### Fluorescence in-situ hybridization (FISH)

Interphase FISH with Vysis dual color break-apart *MYC* (8q24), and dual color break-apart *BCL2* (18q21) probes (Abbott Molecular Inc., Des Plaines, IL) were performed on paraffin-embedded tissue sections. The 5′ and 3′ portions of the *MYC* and *BCL2* probes are labeled with SpectrumOrange and SpectrumGreen, respectively. Interphase FISH analysis with Vysis dual color dual fusion *IGH* (14q32) and *BCL2* (18q21) probes were performed on the lymph node touch imprints. Negative and positive control slides were performed in parallel with the patient sample in the same hybridization process. A total of 100 interphase cells were evaluated for *MYC* and *BCL2* rearrangement independently by two laboratory technologists. An abnormal result was determined if the percentage of cells with abnormal FISH signals (i.e. separation of the 5′ and 3’ *MYC* and *BCL2* probes due to a translocation or abnormal FISH signal patterns due to fusions) was above the relevant cut-off values at 95% confidence.

### Immunoglobulin heavy chain (IGH) clonality assay

Molecular analyses were performed to assess for the presence of clonal IGH rearrangements. DNA was extracted from fresh bone marrow aspirate or formalin-fixed, paraffin-embedded tissue sections. DNA extraction and purification was performed on automated nucleic extraction instrument QIAsymphony SP using QIAsymphony DNA Mini Kit (Qiagen, Valencia, CA). Multiplex PCR-based clonality assays were performed following manufacturers’ protocols (Invivoscribe, San Diego, CA, USA). The PCR products are detected by capillary gel electrophoresis on an ABI 3130XL genetic analyzer (Applied Biosystems, Foster City, CA, USA). An amplification band was considered clonal if the height of the peak was more than 3 times the height of the third highest peak in a given range of DNA sizes.

### *BRAF* V600E mutational analysis

Analysis of *BRAF* V600E mutation was performed using COBAS 4800 *BRAF* Mutation assay according to the manufacturer’s protocol (Roche Molecular Diagnostics). The results were reported as follows: i) V600E mutation detected, ii) V600E mutation not detected, or iii) invalid (ie, no result was obtained on the COBAS test). The COBAS *BRAF* Mutation assay can detect the BRAF V600E mutation at greater than 5% mutation level.

### Whole exome sequencing

Whole exome sequencing was performed at the UCLA Technology Center for Genomics & Bioinformatics. DNA was extracted from the formalin fixed paraffin embedded tissue sections from both the breast biopsy and inguinal lymph node. The library construction was performed using the SeqCap EZ System from NimbleGen (Roche NimbleGen, Inc. Madison, WI) according to the manufacturer’s instructions. Briefly, genomic DNA was sheared, size selected to roughly 300 base pairs, and the ends were repaired and ligated to specific adapters and multiplexing indexes. Fragments were then incubated with SeqCap biotinylated DNA baits after LM-PCR and the hybrids were purified using streptavidin-coated magnetic beads. After amplification of 18 or less PCR cycles, the libraries were then sequenced on the HiSeq 3000 platform from Illumina, using 100-bp pair-ended reads.

The sequence data were aligned to the GRCh37 human reference genome using BWA v0.7.7-r411. PCR duplicates were marked using MarkDuplicates program in Picard-tools-1.115 tool set. GATK v3.2–2 was used for INDEL (insertions and deletions) realignment and base quality recalibration. Exome coverage was calculated using the bedtools. Samtools was used to call the SNVs (single nucleotide variants) and small INDELs. Varscan2 was used to call the somatic SNVs. All variants were annotated using the Annovar program. Copy number alterations and structural variants were not covered in this assay. The variants were ranked by SIFT and PolyPhen scores. A variant with SIFT score < 0.05 is predicted as deleterious (D), otherwise is tolerated (T). A variant with Polyphen score > 0.9 is predicted probably damaging, < 0.45 is predicted benign, between 0.45 and 0.9 is predicted possibly damaging. Variants were filtered out based on allele frequency of > 1% reported in 1000Genome, exAC (The Exome Aggregation Consortium); minimum read-depth of 30; any variants that were in the UTR or upstream/downstream of the gene; variants with a synonymous coding effect and variants within intronic region, unless they result in splice site mutations. Variants of 100% allele frequency in both tissues were considered germline and not included.

### *KRAS* mutational analysis

Evaluation of *KRAS* mutational status was performed using the *KRAS* Mutation real-time PCR Analysis Kit (EntroGen, Tarzana, CA, USA) following the manufacturer’s protocol. This assay employs allele-specific primers that are complementary to mutant variants of the *KRAS* genes. Detection of the amplification products was performed with the use of fluorescent hydrolysis probes. Probes tagged with the FAM fluorophore were complementary to the targets of *KRAS* genes. This assay identifies the following mutations in codon 12: G12S, G12D, G12V, G12C, G12A, G12R; in codon 13: G13D; and in codon 61: Q61H (61CAA > CAT), Q61L, Q61R, Q61H (61CAA > CAC).

### Single nucleotide polymorphism (SNP) microarray analysis

SNP microarray analysis was performed using the OncoScan FFPE Express 3.0 assay kit (Affymetrix, Santa Clara, CA) as per manufacturer’s recommendation. The assay utilizes Molecular Inversion Probe (MIP) technology, which is optimized for highly degraded FFPE samples (probe interrogation site of just 40 base pairs) (1–3). The assay employs 220,000 SNP MIP probes which ensure a resolution of 50–100 kb in selected genomic regions covering 900 cancer genes and of 300 kb outside of these regions. Briefly, 10 unstained FFPE tissue sections cut at 10 μM were obtained and tumor was macrodissected using a hematoxylin and eosin -stained slide as a guide. DNA was extracted and purified from the samples using the QIAmp DNA FFPE Tissue Kit (Qiagen, Dusseldorf, Germany) according to the manufacturer’s protocols. Extracted DNA was quantified using the Quant-iT PicoGreen dsDNA Assay Kit (Invitrogen, Carlsbad, CA, USA) following the manufacturer’s protocol. A total of 80 ng of DNA from each sample was annealed to the MIP probe panel. Annealed MIPs were circularized, followed by enzymatic removal of any un-ligated probe and template DNA. Remaining MIPs were linearized, amplified, enzymatically fragmented, and hybridized to oligonucleotide microarrays. The arrays were washed, scanned, and the results were analyzed and interpreted using OncoScan Console and Nexus Express for OncoScan 3 software (BioDiscovery, El Segundo, CA, USA) as previously described [58–60].

## Additional files

Additional file 1: **Table S1.** Variants identified in both follicular lymphoma and Langerhans cell sarcoma. (DOCX 16 kb)

Additional file 2: **Table S2.** Variants identified in follicular lymphoma. (DOCX 16 kb)

Additional file 3: **Table S3.** Variants demonstrate a loss of heterozygosity during transformation from follicular lymphoma to Langerhans cell sarcoma. (DOCX 16 kb)

Additional file 4: **Table S4.** Variants identified in Langerhans cell sarcoma. (DOCX 17 kb)
