# Supplementary material for: KRAS mutation in secondary malignant histiocytosis arising from low grade follicular lymphoma
Source: Diagn Pathol. 2018 Oct 15;13:78. doi: 10.1186/s13000-018-0758-0 (PMC6190545; doi:10.1186/s13000-018-0758-0)
Supplement: Supplementary file 1 — Table S1. Variants identified in both follicular lymphoma and Langerhans cell sarcoma. (DOCX 16 kb) [file 13000_2018_758_MOESM1_ESM.docx]

**Table S1. Variants identified in both follicular lymphoma and Langerhans cell sarcoma**

| Gene | Exonic Function | Amino acid change | Alternative allele frequency (1000genome)/ExAC | dbSNP | SIFT, PolyPhen | COSMIC | Clinvar | VAF (Follicular lymphoma, Langerhans cell sarcoma |
| --- | --- | --- | --- | --- | --- | --- | --- | --- |
| SPEN | NM_015001:exon11:c.A3191T:p.K1064I | nonsynonymous SNV | na | na | 0.02,0.98,D | na | na | 0.38, 0.49 |
| PDE4DIP | NM_001198834:exon34:c.C5599T:p.R1867C | nonsynonymous SNV | na/na | rs1620560 | 0.01,0.99,D | na | na | 0.51, 0.58 |
| PDE4DIP | NM_001002811:exon6:c.A1718T:p.E573V | nonsynonymous SNV | na/na | rs1061308 | 0,1.00,D | na | na | 0.54, 0.52 |
| PDE4DIP | NM_001002811:exon3:c.C1313T:p.S438L | nonsynonymous SNV | na/na | rs1359300 | 0.01,0.99,D | na | na | 0.46, 0.41 |
| PDE4DIP | NM_001002811:exon1:c.G379A:p.A127T | nonsynonymous SNV | na/na | rs2762745 | 0.02,0.98,D | na | na | 0.46, 0.42 |
| ARHGAP26 | NM_001135608:exon9:c.C877T:p.R293W | nonsynonymous SNV | Na/ 0.00005 | rs369306277 | 0,1.00,D | na | na | 0.47, 0.48 |
| PMS2 | NM_000535:exon2:c.A52G:p.I18V | nonsynonymous SNV | 0.0031 | na | 0.01,0.99,D | COSM601786 | CLINSIG=non-pathogenic | 0.47, 0.51 |
| ATM | NM_000051:exon3:c.C146G:p.S49C | nonsynonymous SNV | 0.0041 | na | 0,1.00,D | na | CLINSIG=other | 0.45, 0.58 |
| KMT2D | NM_003482:exon11:c.C3392T:p.P1131L | nonsynonymous SNV | 0.0002/ 0.0013 | rs201623566 | 0,1.00,D | COSM88089 | CLINSIG=untested | 0.66, 0.39 |
| SPECC1 | NM_001033554:exon5:c.G2062A:p.D688N | nonsynonymous SNV | 0.0078/ 0.0164 | rs35835131 | 0.02,0.98,D | na | na | 0.38, 0.60 |
| ZNF384 | NM_001039920:exon9:c.1153_1155del:p.385_385del | inframeshift deletion | na | na | na | na | na | 0.47, 0.62 |
| CREBBP | NM_001079846:exon29:c.4920_4922del:p.1640_1641del | inframeshift deletion | na | na | na | na | na | 0.31, 0.23 |
