# Supplementary material for: KRAS mutation in secondary malignant histiocytosis arising from low grade follicular lymphoma
Source: Diagn Pathol. 2018 Oct 15;13:78. doi: 10.1186/s13000-018-0758-0 (PMC6190545; doi:10.1186/s13000-018-0758-0)
Supplement: Supplementary file 2 — Table S2. Variants identified in follicular lymphoma. (DOCX 16 kb) [file 13000_2018_758_MOESM2_ESM.docx]

**Table S2. Variants identified in follicular lymphoma**

| Gene | Exonic Function | Amino acid change | Alternative allele frequency (1000genome)/ExAC | dbSNP | SIFT, PolyPhen | COSMIC | Clinvar | VAF (Follicular lymphoma, Langerhans cell sarcoma |
| --- | --- | --- | --- | --- | --- | --- | --- | --- |
| IL2 | NM_000586:exon3:c.G265C:p.V89L | nonsynonymous SNV | na | na | 0.01,0.99,D | na | na | 0.18 |
| PDE4DIP | NM_001198834:exon37:c.A6083G:p.K2028R | nonsynonymous SNV | Na/ 0.0269 | rs139822181 | 1,0.00,T | na | na | 0.27 |
| PDE4DIP | NM_001002811:exon1:c.T248A:p.L83Q | nonsynonymous SNV | Na/ 0.2671 | rs41315685 | 0,1.00,D | na | na | 0.18 |
| CDKN2A | NM_058195:exon1:c.T2C:p.M1T | nonsynonymous SNV | na | na | 0,1.00,D | na | na | 0.15 |
| BCL2 | NM_000657:exon2:c.G608A:p.S203N | nonsynonymous SNV | Na/ 0.000010 | rs377753316 | 0.05,0.95,D | COSM1316823 | na | 0.30 |
| BCL2 | NM_000633:exon2:c.G133A:p.A45T | nonsynonymous SNV | na | na | 0.42,0.58,T | na | na | 0.33 |
| SMARCA4 | NM_001128845:exon18:c.G2648A:p.G883D | nonsynonymous SNV | na | na | 0,1.00,D | na | na | 0.23 |
| KMT2D | NM_003482:exon48:c.C15289T:p.R5097X | stopgain | na | na | 0,1.00,D | COSM221065 | na | 0.21 |
| ERBB2 | NM_001289937:exon17:c.A1963G:p.I655V | nonsynonymous SNV | 0.121406 | rs1136201 | 0.39,0.61,T | na | CLINSIG=non-pathogenic | 0.46 |
| MLLT3 | NM_001286691:exon5:c.435_525del | inframeshift deletion | na | na | na | na | na | 0.13 |
| MN1 | NM_002430:exon1:c.1570_1572del:p.524_524del | inframeshift deletion | na | na | na | na | na | 0.11 |
| MN1 | NM_002430:exon1:c.910_912del:p.304_304del | inframeshift deletion | na | na | na | na | na | 0.12 |
