# Supplementary material for: KRAS mutation in secondary malignant histiocytosis arising from low grade follicular lymphoma
Source: Diagn Pathol. 2018 Oct 15;13:78. doi: 10.1186/s13000-018-0758-0 (PMC6190545; doi:10.1186/s13000-018-0758-0)
Supplement: Supplementary file 3 — Table S3. Variants demonstrate a loss of heterozygosity during transformation from follicular lymphoma to Langerhans cell sarcoma. (DOCX 16 kb) [file 13000_2018_758_MOESM3_ESM.docx]

**Table S3. Variants demonstrate a loss of heterozygosity during transformation from follicular lymphoma to Langerhans cell sarcoma**

| Gene | Amino acid change | Exonic Function | Alternative allele frequency (1000genome)/ExAC | dbSNP | SIFT, PolyPhen | COSMIC | Clinvar | VAF |
| --- | --- | --- | --- | --- | --- | --- | --- | --- |
| PLEKHG5 | NM_001042664:exon13:c.A1318G:p.M440V | nonsynonymous SNV | 0.048123/ 0.0875 | rs61740145 | 0.58,0.42, T | na | na | 0.43 |
| SPOCD1 | NM_001281987:exon2:c.A325G:p.T109A | nonsynonymous SNV | 0.6875/ 0.3178 | rs6664445 | 1,0.00,T | na | na | 0.56 |
| TACSTD2 | NM_002353:exon1:c.G441C:p.E147D | nonsynonymous SNV | 0.0377396/ 0.4881 | rs1062964 | 0.03,0.97,D | na | na | 0.28 |
| IL17RC | NM_001203263:exon4:c.C332T:p.S111L | nonsynonymous SNV | 0.386981/ 0.4881 | rs708567 | 0.2,0.80,T | na | na | 0.47 |
| SLC9C1 | NM_183061:exon11:c.A1270G:p.T424A | nonsynonymous SNV | 0.32488/ 0.3032 | rs6768523 | 0.28,0.72,T | na | na | 0.52 |
| PCDHB8 | NM_019120:exon1:c.A597C:p.K199N | nonsynonymous SNV | 0.0966454/ 0.1419 | rs2950845 | 0.01,0.99,D | na | na | 0.60 |
| KCNH2 | NM_172057:exon7:c.A1670C:p.K557T | nonsynonymous SNV | 0.136182/ 0.1872 | rs1805123 | 0.11,0.89,T | na | CLINSIG=unknown | 0.39 |
| NUGGC | NM_001010906:exon6:c.A538G:p.S180G | nonsynonymous SNV | 0.584465/ 0.4294 | rs4732620 | 0.43,0.57,T | na | na | 0.47 |
| RGS3 | NM_144489:exon1:c.C330G:p.C110W | nonsynonymous SNV | 0.835863/ 0.2143 | rs10817493 | 0.09,0.91,T | COSM4163250 | na | 0.51 |
| KRTAP5-5 | NM_001001480:exon1:c.G50T:p.R17L | nonsynonymous SNV | Na/ 0.2298 | rs66665994 | 0.13,0.87,T | na | na | 0.66 |
| HEATR4 | NM_203309:exon2:c.G41A:p.C14Y | nonsynonymous SNV | 0.0335463/ 0.0414 | rs28552441 | na | na | na | 0.44 |
| PTX4 | NM_001013658:exon3:c.C863A:p.A288D | nonsynonymous SNV | 0.279353/ 0.2330 | rs2745097 | 0.12,0.88,T | na | na | 0.52 |
| KRT32 | NM_002278:exon4:c.G839A:p.R280H | nonsynonymous SNV | 0.179513/ 0.2576 | rs72830046 | 0.05,0.95,D | na | na | 0.60 |
| PREX1 | NM_020820:exon32:c.A4018G:p.K1340E | nonsynonymous SNV | 0.0084/ 0.0225 | rs2664521 | 1,0.00,T | na | na | 0.55 |
