# Supplementary material for: KRAS mutation in secondary malignant histiocytosis arising from low grade follicular lymphoma
Source: Diagn Pathol. 2018 Oct 15;13:78. doi: 10.1186/s13000-018-0758-0 (PMC6190545; doi:10.1186/s13000-018-0758-0)
Supplement: Supplementary file 4 — Table S4. Variants identified in Langerhans cell sarcoma. (DOCX 17 kb) [file 13000_2018_758_MOESM4_ESM.docx]

**Table S4. Variants identified in Langerhans cell sarcoma**

| Gene | Amino acid change | Exonic Function | Alternative allele frequency (1000genome)/ExAC | dbSNP | SIFT, PolyPhen | COSMIC | Clinvar | VAF |
| --- | --- | --- | --- | --- | --- | --- | --- | --- |
| FMN2 | NM_020066:exon5:c.G2822C:p.G941A | nonsynonymous SNV | 0.276558 | rs4997328 | na | na | na | 0.21 |
| BIN1 | NM_139347:exon10:c.C847T:p.H283Y | nonsynonymous SNV | na | na | 0.04,0.96,D | na | na | 0.12 |
| CADM2 | NM_001167674:exon5:c.T653A:p.M218K | nonsynonymous SNV | na | na | 0.9,0.10,T | na | na | 0.12 |
| HDDC2 | HDDC2:NM_016063:exon4:c.C329G:p.T110S | nonsynonymous SNV | na | na | 0.09,0.91,T | na | na | 0.11 |
| TMEM200A | NM_001258276:exon2:c.G1147C:p.G383R | nonsynonymous SNV | na | na | 0.01,0.99,D | na | na | 0.14 |
| ACTL6B | NM_016188:exon8:c.A698G:p.N233S | nonsynonymous SNV | na | na | 0.2,0.80,T | na | na | 0.2 |
| MUC12 | NM_001164462:exon2:c.A3761C:p.K1254T | nonsynonymous SNV | 0.824081 | rs200925391 | 1,0.00,T | na | na | 0.12 |
| FOXD4L3 | NM_199135:exon1:c.T803C:p.L268P | nonsynonymous SNV | na | rs2482272 | 0.15,0.85,T | na | na | 0.26 |
| DNAJB12 | NM_001002762:exon1:c.C37T:p.R13W | nonsynonymous SNV | na | na | 0.02, 0.98,D | na | na | 0.10 |
| SLC22A12 | NM_001276326:exon2:c.C485T:p.A162V | nonsynonymous SNV | na | na | 1,0.00,T | na | na | 0.10 |
| KRAS | NM_004985:exon2:c.G38A:p.G13D | nonsynonymous SNV | na | na | 0,1.00,D | COSM1140132,COSM532 | CLINSIG=pathogenic | 0.24 |
| TRPV4 | NM_147204:exon4:c.G746A:p.R249H | nonsynonymous SNV | na | na | 0.03,0.97,D | na | na | 0.13 |
| EP400 | NM_015409:exon23:c.C4628T:p.A1543V | nonsynonymous SNV | na | na | 0.72,0.28,T | na | na | 0.16 |
| ADAD2 | NM_001145400:exon6:c.G896A:p.R299Q | nonsynonymous SNV | na | na | 0.03,0.97,D | na | na | 0.12 |
| KRTAP4-7 | NM_033061:exon1:c.A337T:p.S113C | nonsynonymous SNV | Na/ 0.0004 | rs9894966 | 0.05,0.95,D | na | na | 0.24 |
| EMR2 | NM_001271052:exon13:c.G1559T:p.C520F | nonsynonymous SNV | na | na | 0,1.00,D | na | na | 0.10 |
| C19orf47 | NM_001256440:exon9:c.C837A:p.D279E | nonsynonymous SNV | na | na | 1, 0.00,T | na | na | 0.14 |
| SHROOM4 | NM_020717:exon4:c.G1410T:p.Q470H | nonsynonymous SNV | na | na | 0.06,0.94,T | na | na | 0.21 |
| ARMCX4 | NM_001256155:exon2:c.G5441A:p.G1814E | nonsynonymous SNV | na | na | na | na | na | 0.12 |
| ARMCX4 | NM_001256155:exon2:c.A5447G:p.E1816G | nonsynonymous SNV | na | na | na | na | na | 0.20 |
| ARMCX4 | NM_001256155:exon2:c.G5459A:p.G1820E | nonsynonymous SNV | na | na | na | na | na | 0.20 |
| HLA-A | NM_001242758:exon5:c.C952T:p.L318F | nonsynonymous SNV | 0.535343/ 0.4748 | rs3179982 | 0,1.00,D | na | na | 0.38 |
| HLA-A | NM_001242758:exon6:c.A1033T:p.T345S | nonsynonymous SNV | 0.686502/ 0.3437 | rs2231119 | 0,1.00,D | na | na | 0.26 |
| KMT2C | NM_170606:exon18:c.G2963T:p.C988F | nonsynonymous SNV | Na/ 0.1065 | rs28522267 | 0,1.00,D | COSM150426 | na | 0.53 |
| KMT2C | NM_170606:exon14:c.C2315T:p.S772L | nonsynonymous SNV | Na/ 0.4861 | rs4024453 | 0.14,0.86,T | COSM4162018 | na | 0.51 |
| NCOR1 | NM_001190438:exon2:c.C188T:p.S63L | nonsynonymous SNV | Na/ 0.4731 | rs150910818 | 0.72,0.28,T | na | na | 0.32 |
| CAMTA1 | NM_015215:exon16:c.3813dupT:p.N1271fs | frameshift insertion | na | na | na | na | na | 0.11 |
| ARID1A | NM_006015:exon16:c.3999_4001del:p.1333_1334del | nonframeshift deletion | na | na | na | COSM133030 | na | 0.09 |
| NOTCH1 | NM_017617:exon34:c.7231_7233del:p.2411_2411del | nonframeshift deletion | na | na | na | COSM27388 | na | 0.15 |
| BCR | NM_021574:exon18:c.3146_3147insCCGG:p.G1049fs | frameshift insertion | na | na | na | na | na | 0.22 |
